# M6A-related bioinformatics analysis indicates that LRPPRC is an immune marker for ischemic stroke

**DOI:** 10.1038/s41598-024-57507-y

**Published:** 2024-04-17

**Authors:** Lianwei Shen, Shouwei Yue

**Affiliations:** Rehabitation Center, Qilu Hospital of Shandong University, No. 107, West Culture Road, Lixia District, Jinan, 250012 Shandong China

**Keywords:** Ischemic stroke, N6-Methyladenosine modulation, Predictive model, Immunity, Cluster, Computational biology and bioinformatics, Immunology

## Abstract

Ischemic stroke (IS) is a common cerebrovascular disease whose pathogenesis involves a variety of immune molecules, immune channels and immune processes. 6-methyladenosine (m6A) modification regulates a variety of immune metabolic and immunopathological processes, but the role of m6A in IS is not yet understood. We downloaded the data set GSE58294 from the GEO database and screened for m6A-regulated differential expression genes. The RF algorithm was selected to screen the m6A key regulatory genes. Clinical prediction models were constructed and validated based on m6A key regulatory genes. IS patients were grouped according to the expression of m6A key regulatory genes, and immune markers of IS were identified based on immune infiltration characteristics and correlation. Finally, we performed functional enrichment, protein interaction network analysis and molecular prediction of the immune biomarkers. We identified a total of 7 differentially expressed genes in the dataset, namely METTL3, WTAP, YWHAG, TRA2A, YTHDF3, LRPPRC and HNRNPA2B1. The random forest algorithm indicated that all 7 genes were m6A key regulatory genes of IS, and the credibility of the above key regulatory genes was verified by constructing a clinical prediction model. Based on the expression of key regulatory genes, we divided IS patients into 2 groups. Based on the expression of the gene LRPPRC and the correlation of immune infiltration under different subgroups, LRPPRC was identified as an immune biomarker for IS. GO enrichment analyses indicate that LRPPRC is associated with a variety of cellular functions. Protein interaction network analysis and molecular prediction indicated that LRPPRC correlates with a variety of immune proteins, and LRPPRC may serve as a target for IS drug therapy. Our findings suggest that LRPPRC is an immune marker for IS. Further analysis based on LRPPRC could elucidate its role in the immune microenvironment of IS.

## Introduction

Currently, regarding the treatment of ischemic stroke, in addition to the classical intravenous thrombolytic therapy, the addition of endovascular treatment with mechanical thrombectomy and acute reperfusion therapy have also been proven to be effective^1^. However, all of these treatments have limitations. The effective therapeutic window for intravenous thrombolysis is only 3 h^2^. The addition of mechanical thrombectomy to endovascular therapy is only indicated for proximal intracranial vascular occlusion^3^. Acute reperfusion therapy requires a high level of therapeutic team and specialized equipment^3^. Thus, it is necessary to find new therapeutic approaches. In recent years, the role of RNA modification in gene regulation has received extensive attention from the academic community. N6-methyladenosine (m6A) modification is the most common means of mRNA modification in eukaryotes. It can modulate mRNA immune metabolism and immune processes by affecting transcript stability, splicing, translation efficiency and hat non-dependent translation. It plays a key role in immune metabolism and immunologic processes in cancer, neurological diseases and metabolic diseases^4,5^. Among the more than 150 RNA modification methods identified so far, methylation is the most abundant^6^. It has been demonstrated that m6A-related genes may exist as therapeutic targets and diagnostic biomarkers for IS, and are involved in the immune regulation of stroke occurrence and development^7^. Therefore, the present study used a bioinformatics approach to comprehensively analyze the role of genes related to the regulation of m6A modifications in immune infiltration in ischemic stroke. The aim was to identify immune markers of IS and further explore immune molecules and/or immune-related drugs that may be associated with immune markers.

## Methods

### Data collection and processing

**Figure 1:**
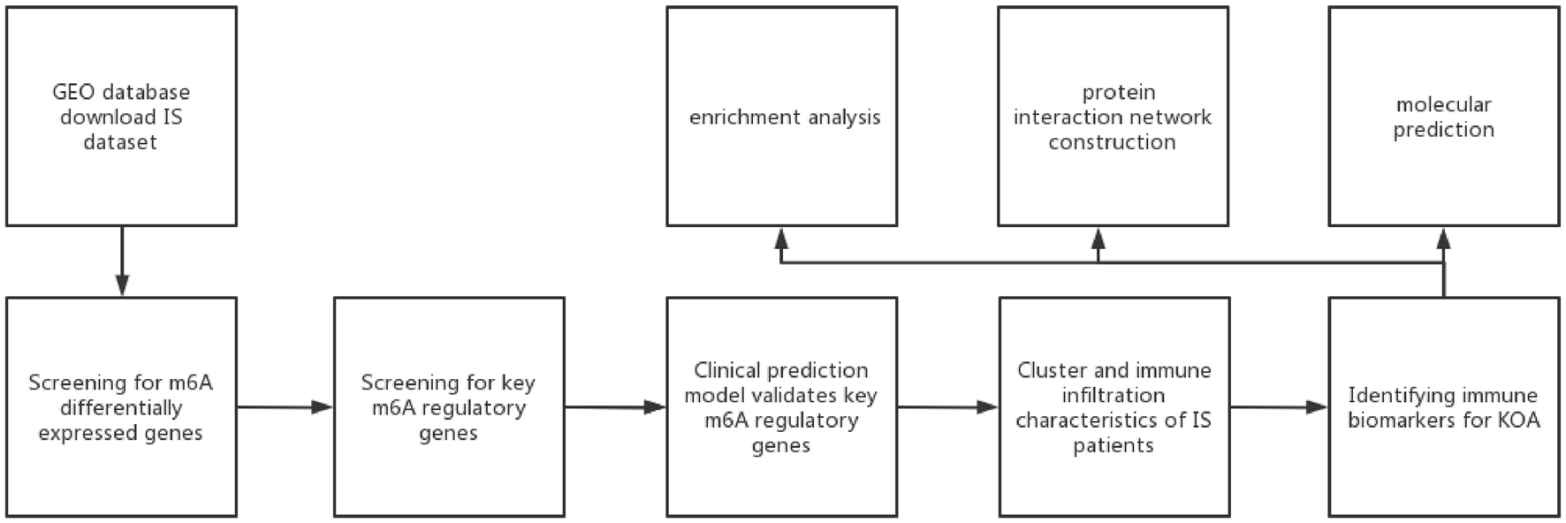
Flowchart.

In this study, RNA expression profiles and clinical information for the ischemic stroke data set GSE58294 were downloaded via the GEO database, which includes blood RNA testing data from 69 ischemic stroke samples due to cardiogenic embolism and 22 controls^8^. These samples were sequenced by the GPL570 (Affymetrix Human Genome U133 Plus 2.0 Array) platform. The expression matrix was normalised using the “normalizeBetween-Arrays” function of the “limma” package in R. The gene probes were annotated with official symbols. The flowchart of the experiment is shown in Fig. 1.

### Screening for m6A-regulated differential genes in IS patients

Based on the current research on m6A, 28 m6A-related genes were included in this study as subjects, including METTL3, METTL14, METTL16, WTAP, VIRMA, ZC3H13, RBM15, RBM15B, CBLL1, YWHAG, TRA2A, CAPRIN1, YTHDC1, YTHDC2, YTHDF1, YTHDF2, YTHDF3, HNRNPC, FMR1, LRPPRC, HNRNPA2B1, IGFBP1, IGFBP2, IGFBP3, RBMX, ELAVL1, IGF2BP1, FTO, and ALKBH5^9,10^. The corresponding positions of the m6A-related genes in the chromosomes were determined by perl software, and the corresponding positions of the m6A-related genes in the chromosomes were visualized by using the “RCircos” package in R language. The expression of m6A-related genes in each sample was extracted using the R software package “limma”. Then Wilcoxon test was used to detect the difference between ischemic stroke patients and controls in the above m6A-related gene expression model, and the difference in m6A-related genes was screened as statistically significant at *P* < 0.05.

### Machine learning-based screening of m6A key regulatory genes

In this study, two widely used machine learning algorithms, random forest (RF) and support vector machine (SVM), were selected. The residual values of m6A differentially regulated genes in the two machine learning algorithms and the inverse distribution of the residuals were plotted by using the “randomForest” package in R, so as to select a suitable model, and then screen out the m6A key regulatory genes based on this model.

### Construct and test clinical prediction models based on m6A key regulatory genes

The “datadist” function in the “rms” package of the R language was used to package the m6A key regulatory genes screened by the model, and the “lrm” function was used to fit the model. The “lrm” function was used to fit the clinical prediction model. Use the “nomogram” function to visualise the clinical prediction model in the form of nomogram. Evaluation of clinical prediction models: C-index was used for differentiation assessment; calibration curve was used for consistency assessment; clinical decision curve and clinical benefit curve were used for patient benefit assessment.

### Classification of IS patients in the dataset based on m6A key regulatory genes

Consensus cluster analysis of IS patients based on m6A key regulatory genes. The number of clusters in the IS samples was determined in R using the “ConsensusClusterPlus” package. The parameters are: “maxK” = 10, “reps” = 100, “pItem” = 0.8, “pFeature” = 1, “clusterAlg” = “hc,” and “distance” = “euclidean”^11^. IS patients were repeatedly sampled 10 times according to the m6A key regulatory genes, and IS patients were classified into 10 different subgroups. The optimal number of clusters was selected based on Calinski’s criterion and correlation between subgroups. The “Rtsne” package was used to display the sample distribution of different clusters. The expression of m6A key regulatory genes between different clusters was compared using the Kruskal–Wallis test in R language.

### Determination of IS immune marker

Single sample gene set enrichment analysis (ssGSEA) was performed using the “gsva” package in R to analyse the immune infiltration of the samples. Twenty-three immune cell types were selected for this study, including Activated.B.cell, Activated.CD4.T.cell, Activated.CD8.T.cell, Activated.dendritic.cell, CD56bright.natural.killer. cell, CD56dim.natural.killer.cell, Eosinophil, Gamma.delta.T.cell, Immature.B.cell, Immature.dendritic.cell, MDSC, Macrophage, Mast.cell, Monocyte, Natural.killer.T.cell, Natural.killer.cell, Neutrophil, Plasmacytoid.dendritic.cell, Regulatory.T.cell, T.follicular.helper. cell, Type.1.T.helper.cell, Type.17.T.helper.cell, and Type.2.T.helper.cell. Infiltrating immune cell abundance scores in two different patient clusters were compared in R using the Kruskal–Wallis test. Heatmaps were created with the “pheatmap” package to show the correlation between the 2 m6A key regulatory genes and these immune cells to determine the identification of IS immunomarkers.

### Enrichment analysis, protein–protein interaction network analysis and molecular prediction of IS immune marker

In this study, we used the “limma” package in R to screen for differential genes between different clusters with |log2 (fold change)| > 1, P < 0.05, and performed enrichment analysis for IS immune marker. Gene ontology (GO) enrichment analysis: The GO database information was referenced through the clusterProfiler package of R and the org.hs.eg/.dbpackage of R. The “enrichplot” package, “ggplot” package, “ggplot” package, “ggplot” package and “ggplot” package were used for the enrichment analysis. GO terms that satisfy this condition are defined as those that are significantly enriched in differentially expressed genes. The top hits with the most significant enrichment (lowest p-value) are shown in the histogram^12^. The STRING database was used to construct protein interaction network analysis (PPI) for immune marker. the more connections in the network, the more important the protein is. STITCH (Search Tool for Interactions of Chemicals) database was used for molecular prediction of immune markers. The database is an online resource focusing on molecular interactions and network drug discovery, aiming to integrate data on biological compounds, proteins, genes, metabolic pathways and many more, and digitally display their interaction and signal networks^13^.

### Ethical approval and participation consent

The data of the human part of the study were obtained from GEO database, and the original study has completed the ethical audit.

## Results

### m6A differential expression gene screening for IS

**Figure 2:**
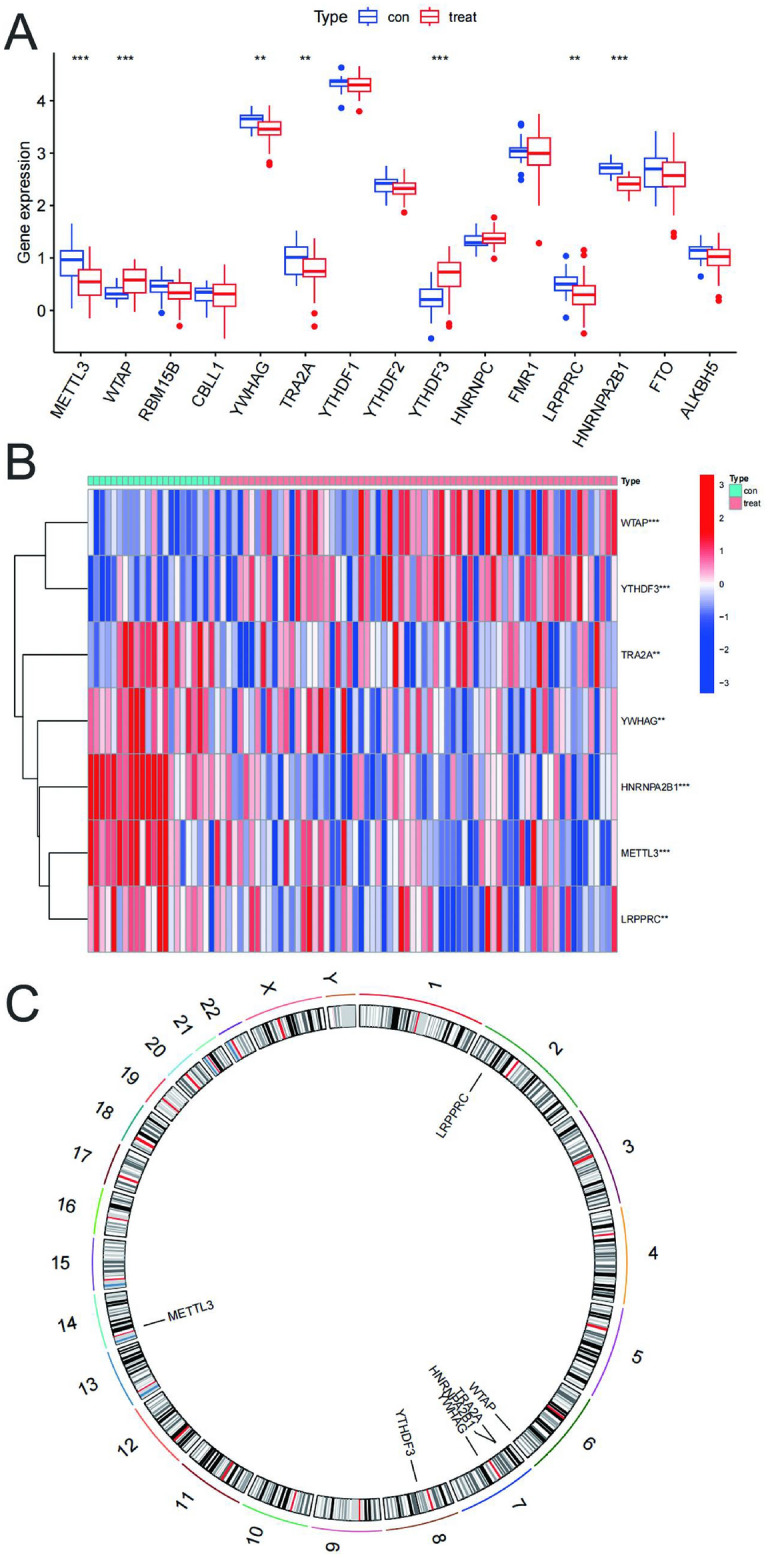
m6A differential expression gene screening for IS. (**A**) Boxplot of 15 m6A genes expression between control and IS. **p < 0.01, ***p < 0.001. (**B**) Heatmap of 7 differentials expressed m6A genes between control and IS Red represents. high expression and blue represents low expression. (**C**) Chromosomal location of m6A differentially expressed genes.

By analysing the GSE58294 data set, we identified METTL3, WTAP, RBM15B, CBLL1, YWHAG, TRA2A, YTHDF1, YTHDF2, YTHDF3, HNRNPC, FMR1, LRPPRC, HNRNPA2B1, FTO, and ALKBH5, and the expression of 15 m6A-associated genes. 7 genes, including METTL3, WTAP, YWHAG, TRA2A, YTHDF3, LRPPRC, and HNRNPA2B1, showed statistical differences. genes were statistically different. Among them, METTL3, YWHAG, TRA2A, LRPPRC, and HNRNPA2B1 were significantly down-regulated, while WTAP, and YTHDF3 were significantly up-regulated (Fig. 2A,B). Meanwhile, we marked the locations of the seven differentially expressed genes on the chromosomes (Fig. 2C).

### Machine learning-based screening of m6A key regulatory genes

**Figure 3:**
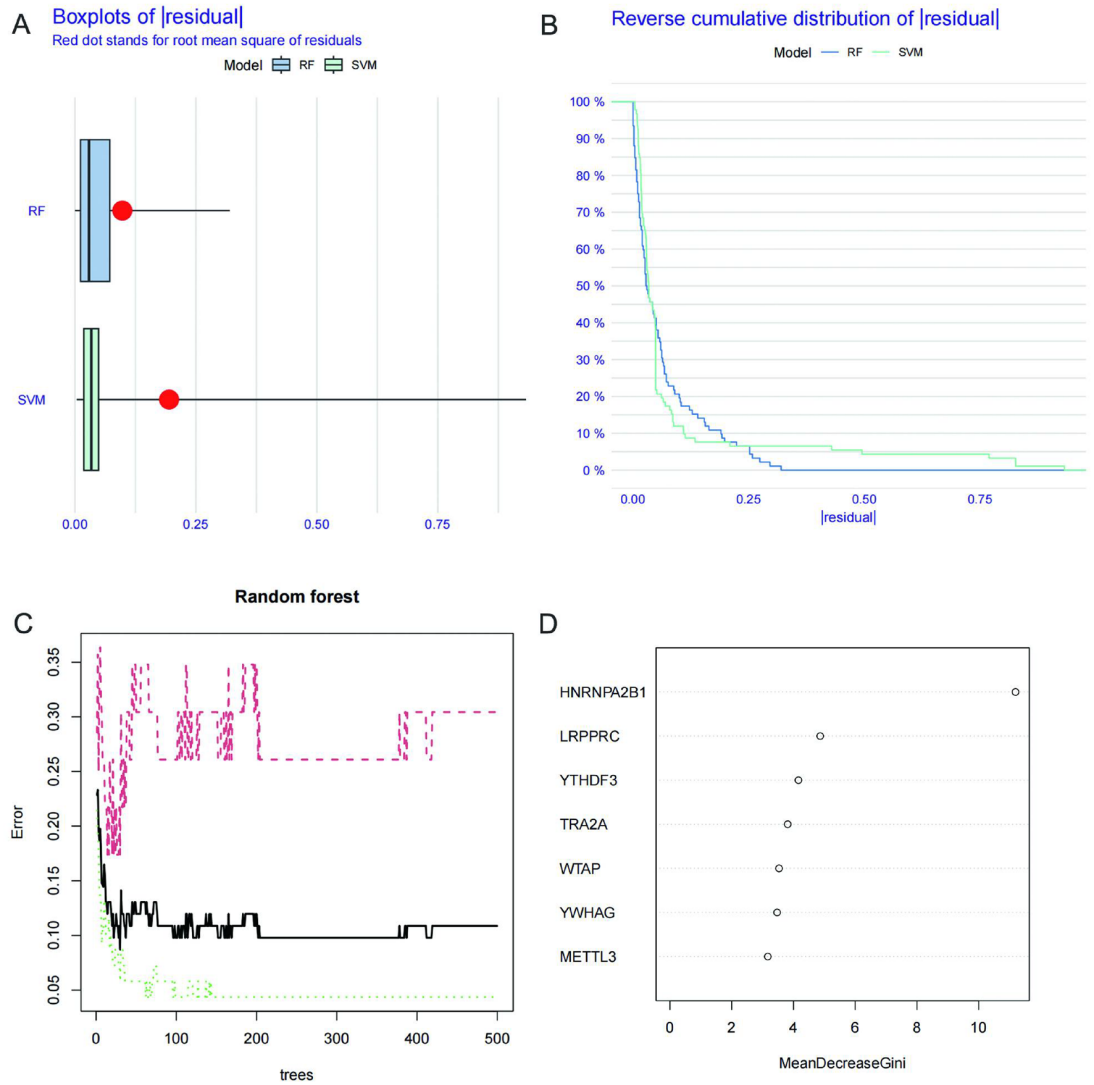
Machine learning-based screening of m6A key regulatory genes. (**A**) Boxplot of residual in RF and SVM. (**B**) Reverse cumulative distribution of residual in RF and SVM. (**C**) RF screening of m6A key regulatory genes. (**D**) Screening for candidate m6A-regulated genes by RF.

We compared the two machine learning algorithms by calculating the residual value and the inverse cumulative distribution graph. The RF algorithm outperformed the SVM in terms of the residual value and the inverse cumulative distribution graph (Fig. 3A,B), so we chose the RF algorithm to be used for screening the key regulatory genes. From the RF graph, it can be seen that the RF algorithm has the smallest error when the tree number is 30 (Fig. 3C). Since the importance scores of the differential genes were all greater than 2, METTL3, WTAP, YWHAG, TRA2A, YTHDF3, LRPPRC, and HNRNPA2B1 were used as key regulatory genes (Fig. 3D).

### Construction and validation of clinical prediction models

**Figure 4:**
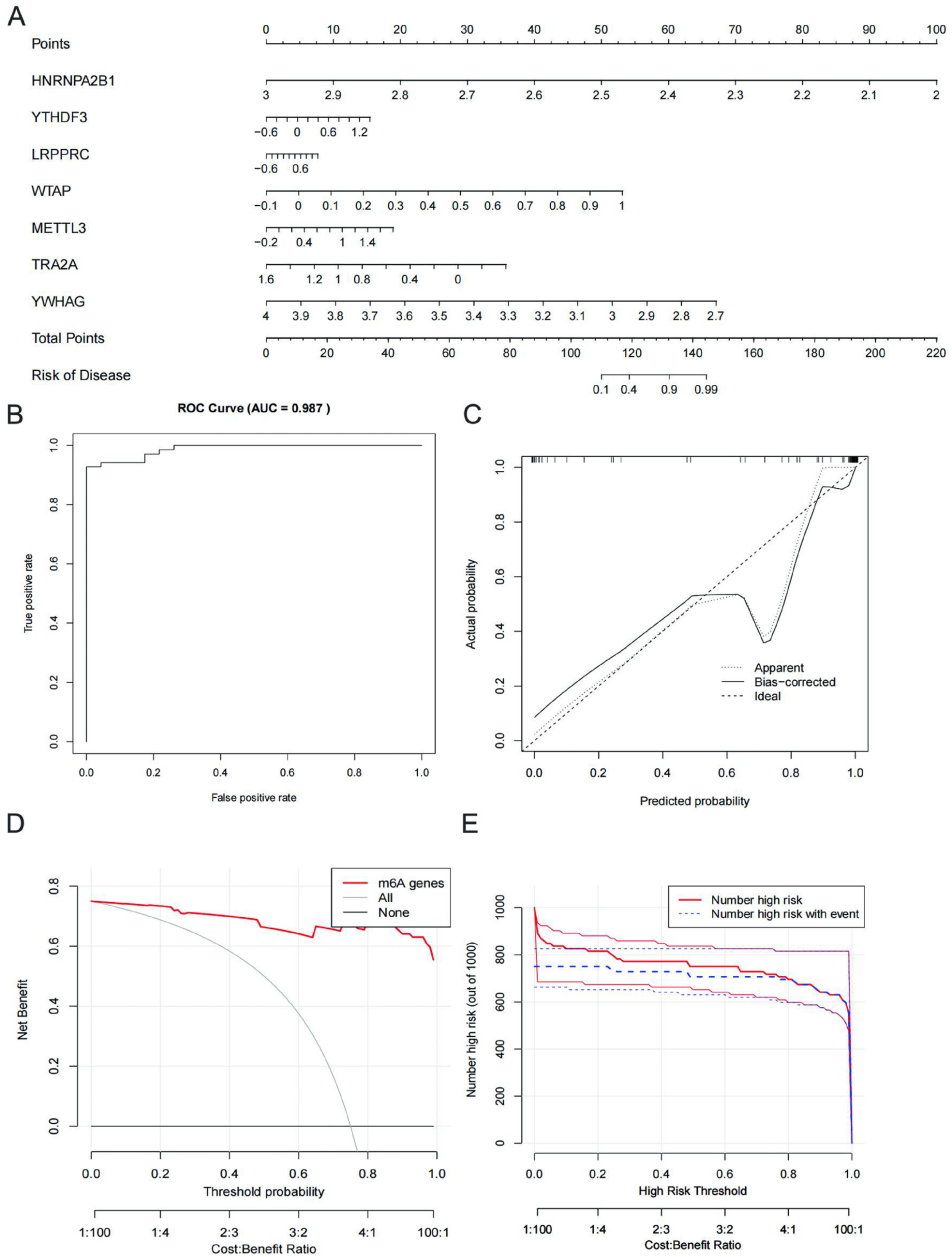
Construction and validation of clinical prediction models. (**A**) Nomogram of m6A key regulatory genes for predicting IS. (**B**) C_index of the model. (**C**) Calibration curve of the model. (**D**) Decision curves for clinical prediction models. (**E**) Benefit curves for clinical prediction models.

We constructed a clinical prediction model using the “lrm” function in the “rms” package, which can be used to assess the correlation between five key regulatory genes and the risk of IS, and visualised the model by a line graph (Fig. 4A). The C-index of the model was 0.987, indicating that the model had good discrimination (Fig. 4B). The calibration of the model was done using Bootstrap self-sampling method by setting the number of resamples to be 1000, and the resulting calibration curve plotted indicates that the model is well calibrated (Fig. 4C). The decision analysis curve and the benefit curve show that the constructed model has good application value (Fig. 4D,E).

### IS patient clusters obtained based on m6A key regulatory genes

**Figure 5:**
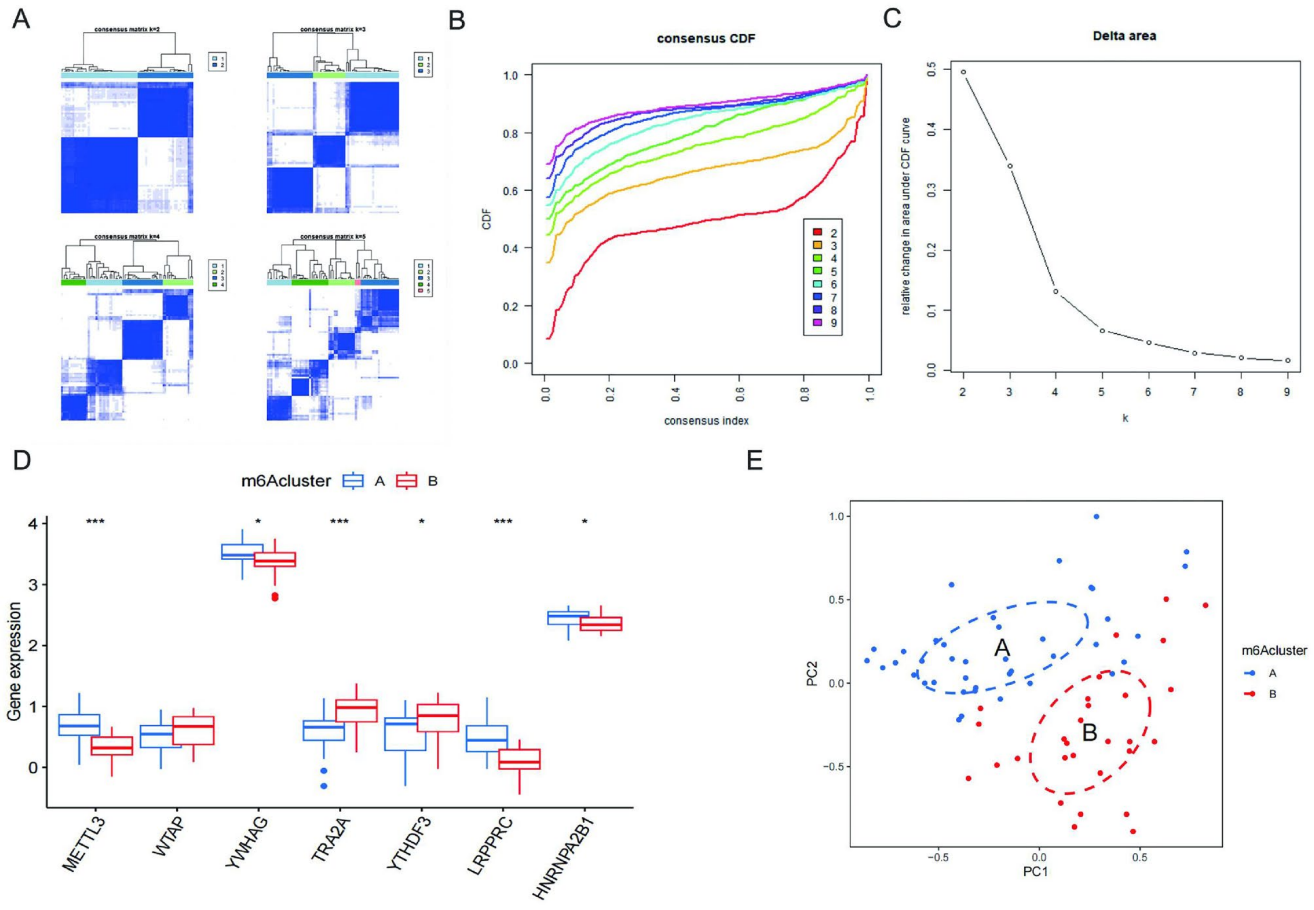
IS patient clusters obtained based on m6A key regulatory genes. (**A**) Consensus clustering matrix of IS samples for k = 2 to k = 5. (**B**) Consensus clustering CDF for k = 2 to k = 9. (**C**) Calinski criterion analysis of IS samples. Calinski criterion optimal number of clusters: 2. (**D**) Boxplot of 5 m6A genes expression in group A and B *p < 0.05, ***p < 0.001. (**E**) PCA analysis between clusters.

Based on the 2 m6A key regulatory genes, we performed a cluster analysis of IS patients. Consensus clustering matrix is shown in Fig. 5A,B. Using the relevance of the subgroups as a criterion, we chose to classify the patients into 2 clusters (Fig. 5B,C). Based on the 2 clusters mentioned above, we extracted the expression profiles of m6A key regulatory genes in different clusters, and the box plots indicated that the expression levels of METTL3, YWHAG, TRA2A, YTHDF3, LRPPRC, and HNRNPA2B1 were significantly different in different clusters (Fig. 5D). Principal component analysis (PCA) showed that the above cluster analysis method could accurately distinguish IS (Fig. 5E). Therefore, the clustering of IS patients based on m6A key regulatory genes in this study was accurate.

### Determination of IS immune marker

**Figure 6:**
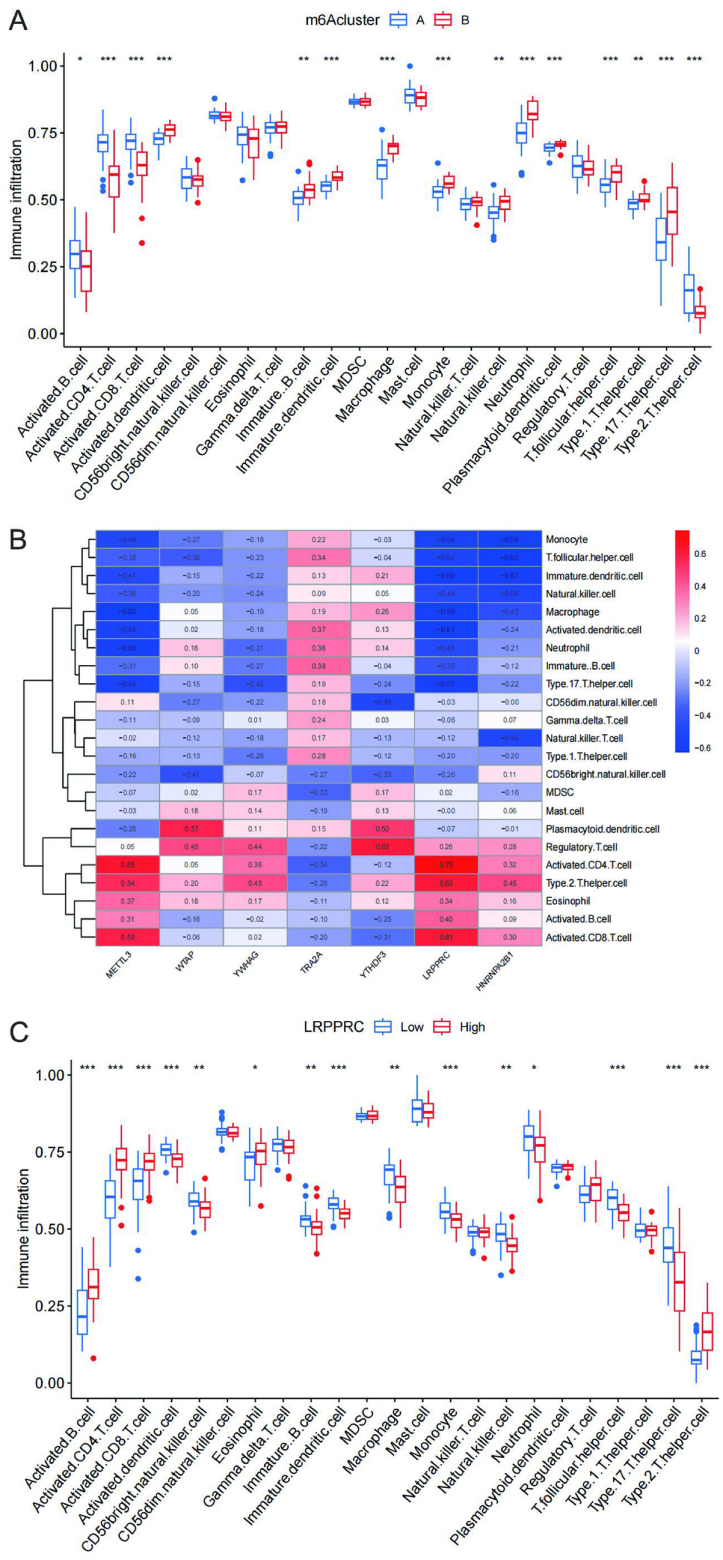
Determination of IS immune marker. (**A**) Differences in immune infiltration abundances in five m6A clusters. *p < 0.05, **p < 0.01, ***p < 0.001. (**B**) Immune cell infiltration correlation heat map of m6A key regulatory genes. (**C**) Immune infiltration analysis between clusters with different LRPPRC expression levels Group Low and group High represent cell clusters with low and high LRPPRC expression, respectively. *p < 0.05, **p < 0.01, ***p < 0.001.

We assessed the level of immune cell infiltration between different clusters by the ssGSEA algorithm to explore the differences in the immune microenvironment of different clusters. Through statistical analysis, we found significant differences in 19 immune cell infiltration types across 2 clusters (Fig. 6A). Meanwhile, we calculated the correlation between the 7 m6A key genes and immune cell infiltration (Fig. 6B), among which the correlation of LRPPRC was the most obvious, with the maximum positive correlation coefficient of 0.75 and the maximum negative correlation coefficient of -0.61.Therefore, LRPPRC may play a key role in immune cell infiltration. As shown in Fig. 6C, we found that by comparing the LRPPRC expression of immune-infiltrating cells of the samples, Activated.B.cell, Activated.CD4.T.cell, Activated.CD8.T.cell, Activated.dendritic.cell, CD56dim.natural.killer.cell, Eosinophil, Immature.B.cell, Immature.dendritic.cell, Macrophage, Monocyte, Natural.killer.cell, Neutrophil, T.follicular.helper.cell, Type.17.T.helper.cell and Type.2.T.helper.cell, and 15 other immune-infiltrating cells were statistically different, suggesting that LRPPRC is an important player in the immune microenvironment of IS, and can be considered as an immune marker for IS.

### Enrichment analysis, PPI network analysis and molecular prediction of LRPPRC

**Figure 7:**
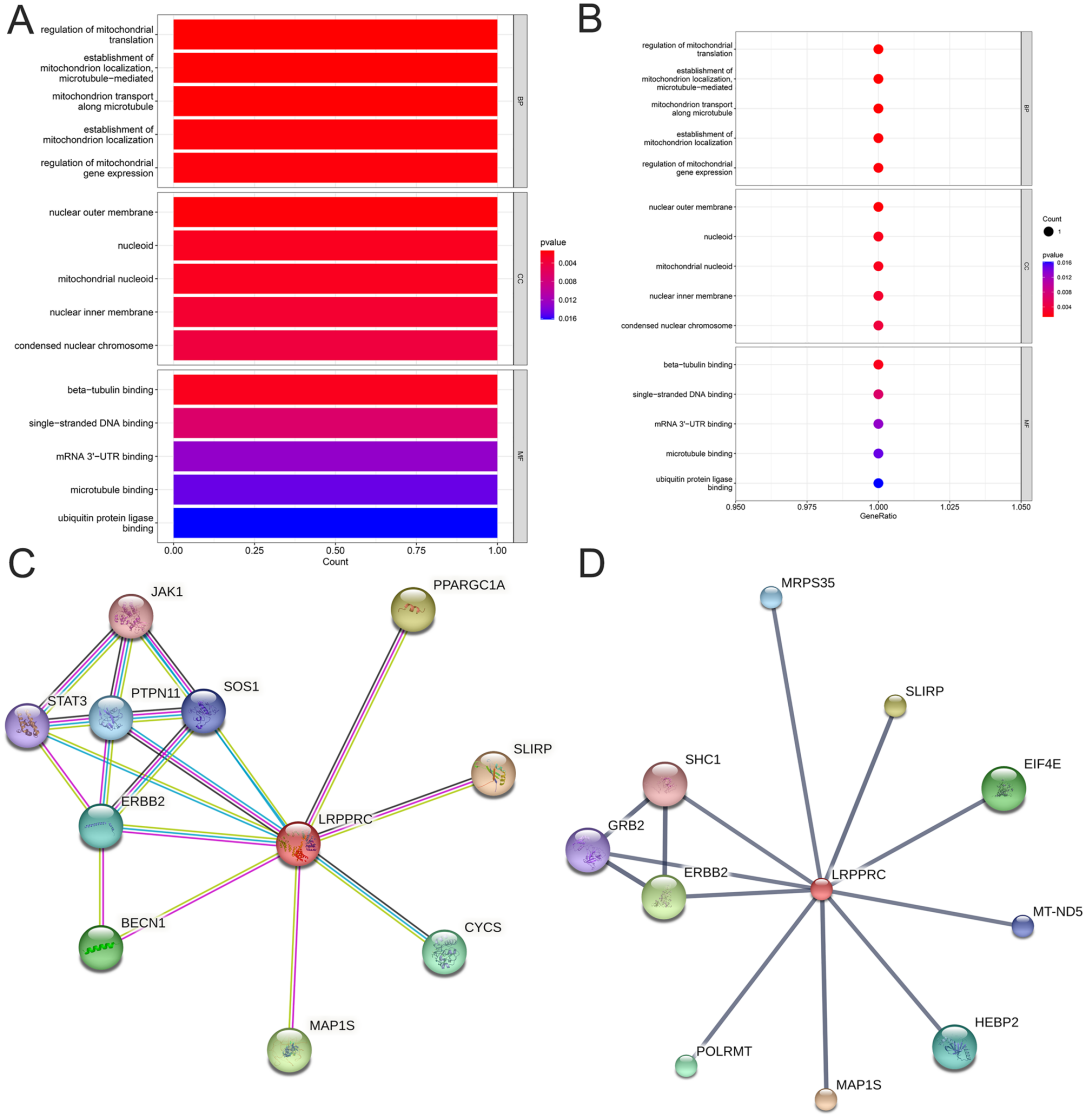
Enrichment analysis, PPI network analysis and molecular prediction of LRPPRCs. (**A**,**B**) Gene Ontology analysis points out the enrichment degree in BP, CC, and MF. (**C**) PPI of LRPPRC. (**D**) Molecular prediction of LRPPRC.

We performed GO enrichment analysis of LRPPRC in R language (Fig. 7A,B). LRPPRC was enriched in mitochondrial function in BP; localised in mitochondria and nucleus in CC; and associated with transcription-translation in MF. We performed molecular prediction in the STRING database indexed by LRPPRC, set the minimum required interaction score as the highest confidence (0.900), and obtained the related molecules and drugs as PPARGC1A, SLIPR, CYCS, MAP1S, BECN1, ERBB2, PTPN11, JAK1, SOS1 and STAT3 (Fig. 7C). We performed protein interaction network analysis in the STITCH database indexed by LRPPRC, set the minimum required interaction score as the highest confidence (0.900), and obtained the interacting proteins as MPRS35, SLIRP, EIF4E, MT-ND5, HEBP2, MAP1S, POLRMT, ERBB2, SHC1 and GRB2 (Fig. 7D).

## Discussion

The development of IS is extremely complex and involves a variety of molecular mechanisms and methylation modifications. M6A modifications are very common in mammalian brain tissues and play an important role in synaptic plasticity, learning, memory and other aspects related to neurological functions. M6A expression abnormality may be one of the causes of a variety of neurological disorders, and m6A-related genes can be found in Alzheimer’s disease, Parkinson's disease, and major depression^5^. Whereas IS can affect the entire brain and its neural network properties, it has been shown that stroke significantly increases RNA methylation in ischemic stroke brain tissue^14^. Current studies on the role of m6A in the development of IS mainly focus on immune-related aspects. m6A modifications can promote microglia/macrophage activation and polarisation and plays a regulatory role in microglia-induced inflammatory responses after stroke^15^. M6A modifications are also closely related to the generation of immune inflammation and alterations of the immune microenvironment after IS^16^, but the development of IS may also be associated with excitotoxicity, ionic imbalance, oxidative stress, endoplasmic reticulum stress, and apoptosis^17^. The exact principle of its action is still unclear. Therefore, it is necessary to use bioinformatics to comprehensively explore the role of m6A regulation in the immunity of IS. We screened five key m6A-regulated genes in IS, in which the expression of METTL3, LRPPRC and HNRNPA2B1 was increased, and the expression of WTAP and YTHDF3 was decreased. M6A methylation mediated by METTL3 promotes the maturation of miR-335, which facilitates the formation of stress granules in the early phase of acute ischaemic stroke^18^. Stress granules can immediately and transiently block mRNA translation to protect valuable mRNAs and proteins from harmful environments, thereby reducing neuronal damage and apoptosis^18^. In a study on IS and immune microenvironment regulation, researchers found that LRPPRC could inhibit the immune response during the development of IS by suppressing dendritic cell activation, thereby attenuating the immune suppression due to neurological deficits and alterations in the systemic immune system, and decreasing the incidence of infections in IS patients^15^. WTAP is an essential bridging protein that stabilises the METTL3-METTL14 complex^19^, thereby promoting the formation of stress granules and protecting neurons. In a study exploring the relationship between m6A and circRNAs in mouse cerebral ischemia, the expression of YTHDF3 decreased and then increased, while the expression of METTL3 decreased. Meanwhile, METTL3 and YTHDF3 may not only act on post-transcriptional regulation of mRNAs only, but also on circRNAs^20^. CircRNAs are closely associated with stroke severity and inflammatory response and are neurospecific, which play key roles in stroke diagnosis, prognosis, and treatment, and may be used as new diagnostic and prognostic biomarkers^21^. After that, we chose machine learning and clinical prediction models for screening of m6A key genes and prediction of the risk of IS occurrence. The RF algorithm can be used not only for prediction of early neurological deterioration in patients with acute mild stroke^22^, but also for prediction of long-term prognosis of IS^23^. We not only found that the residual values and area under the AUC curve of the RF algorithm were superior to the SVM algorithm. the RF algorithm identified METTL3, LRPPRC, HNRNPA2B1, WTAP and YTHDF3 as key regulatory genes for IS. Clinical prediction models can predict the risk of IS occurrence based on m6A key regulatory genes. Because of its simplicity and ease of understanding, column charts have become a common visual representation of clinical prediction models. IS column charts often select the lifestyle habits, past medical history, imaging tests, laboratory tests, etc. of IS patients as the influencing factors of IS to predict the occurrence and development of IS^24,25^. Clinicians can calculate the probability of IS based on the expression of METTL3, LRPPRC, HNRNPA2B1, WTAP and YTHDF3. To further search for biomarkers of IS, we divided the 69 IS patients into 5 clusters based on the differences in the expression of key m6A genes and analyzed the differences in immune infiltration in different clusters. Clustering of patients based on certain information about the patient that is relevant to the target disease is common in clinical practice. In a study on Parkinson's disease, investigators found that baseline clinical typing of patients based on their serum markers predicted their motor or non-motor prognosis, which in turn provided clinicians with ideas for earlier interventions^26^. In a 63-year follow-up study of dementia, researchers found significant differences in the incidence of dementia after clustering the follow-up population by genetic risk of dementia^27^. In our study, we first clustered the IS patients in the dataset based on the m6A key gene, which was known to distinguish IS patients by PCA. In a study of m6A modification in inflammatory bowel disease, clustering of patients also showed different immunophenotypes^28^. To further identify immune markers for m6A, we used the ssGSEA algorithm to look at the level of immune cell infiltration between m6A clusters and identified by immune correlation that LRPPRC could serve as an immune marker for IS. After that, we looked at the immune infiltration characteristics of IS patients grouped based on the expression of LRPPRC and found that LRPPRC plays an important role in the immune microenvironment of IS. Finally, we performed enrichment analysis, PPI and molecular prediction of LRPPRC. LRPPRC may influence stroke development in mitochondrial function, transcriptional translation associated with m6A, and via immune-related proteins^29^. The influenza A virus PB2 protein can block JAK1/STAT signalling by targeting JAK1 for degradation via a proteasome mechanism. This allows the PB2 protein to evade the body’s innate immunity to the virus^30^. Metabolomics and proteomics have shown that SOS1 can serve as a potential biomarker for early IgA nephropathy and is involved in the regulation of immune system activation in IgA patients^31^. STAT3, a transcriptional regulator, plays a key role in vertebrate immunity. Mutations in this factor are associated with immunodeficiency and autoimmune diseases^32^. MAP1S is a protein involved in autophagy and is mainly expressed in macrophages. And MAP1S-deficient macrophages have attenuated phagocytosis of bacteria, thus attenuating the body’s innate immunity to microbial infections^33^. Mutations in ERBB2 promote PD-L1-mediated immune escape in gallbladder cancer^34^. CYCS is significantly associated with immune cells and promotes the proliferation of asthma cells in vitro^35^. Meanwhile, we also identified immune-related molecules by molecular prediction. Over expression of mitochondrial RNA polymerase is associated with abnormal clinical pathology conditions in patients with lung adenocarcinoma, which can lead to decreased life expectancy. Also, mitochondrial RNA polymerase expression is positively correlated with immune suppressor gene, and POLRMT over expression in lung adenocarcinoma patients affects the immune microenvironment of the tumour^36^. A comprehensive analysis of multiple oncology databases found that SHC1 plays an important role in the tumour immune microenvironment. SHC1 has prognostic and diagnostic value in a variety of cancers and may serve as a potential biomarker for cancer immunotherapy and diagnosis^37^. In melanoma, genetic ablation of phosphorylated-eIF4E reprogrammes the immunosuppressive microenvironment, which reduces the production of inflammatory factors and immunoproteins^38^. In summary, LRPPRC plays an important role in the immune microenvironment of IS and may serve as an immune marker for IS. Our study also has its limitations. We should increase m6A-seq sequencing data for IS to fully elucidate the role of m6A in IS^39^.

## Conclusion

In this study, we comprehensively demonstrated the potential of LRPPRC as an immune marker for IS by constructing multiple bioinformatics analyses, which can be used as an idea for further research on drug targets for IS.

## Data Availability

The dataset for this study is GSE58294 from the GEO database, which contains transcriptome-wide data on blood from 69 cardioembolic ischaemic stroke samples and 22 control samples. For more information, visit https://www.ncbi.nlm.nih.gov/geo/query/acc.cgi?acc=GSE58294. This dataset is publicly available.

## Abbreviations

m6A: M6-methyladenosine
IS: Ischemic stroke
SVM: Support vector machines
RF: Random forest
RT-qPCR: Real-time quantitative PCR
PCA: Principal component analysis
STITCH: Search Tool for Interactions of Chemistry
BP: Biological process
CC: Cellular component
MF: Molecular function
ssGSEA: Single sample gene set enrichment analysis
GO: Gene ontology
PPI: Protein interaction network analysis
